# Children's Eating Behavior Questionnaire Correlated with Body Compositions of Thai Children and Adolescents with Obesity: A Pilot Study

**DOI:** 10.1155/2021/6496134

**Published:** 2021-01-15

**Authors:** Ekkarit Panichsillaphakit, Yuda Chongpison, Puthita Saengpanit, Tanisa Kwanbunbumpen, Jaraspong Uaariyapanichkul, Sirinuch Chomtho, Chitsanu Pancharoen, Chonnikant Visuthranukul

**Affiliations:** ^1^Division of Nutrition, Department of Pediatrics, King Chulalongkorn Memorial Hospital, The Thai Red Cross Society, Bangkok 10330, Thailand; ^2^Center for Excellence in Biostatistics, Research Affairs, Faculty of Medicine, Chulalongkorn University, Bangkok 10330, Thailand; ^3^Pediatric Nutrition Research Unit, Division of Nutrition, Department of Pediatrics, Faculty of Medicine, Chulalongkorn University, Bangkok 10330, Thailand; ^4^Division of Infection, Department of Pediatrics, Faculty of Medicine, Chulalongkorn University, Bangkok 10330, Thailand

## Abstract

**Introduction:**

Obesity is a major threat to public health. Eating behavior and dietary intake of especially high energy-dense food with low nutrients contribute to the current epidemic of childhood obesity. However, the relationship between eating behavior and body composition has yet to be examined in Thai children and adolescents with obesity. We assessed the association between children's eating behaviors and their body composition in prerandomized patients who participated in the randomized trial titled “Impact of Dietary Fiber as Prebiotics on Intestinal Microbiota in Obese Thai Children”.

**Methods:**

During the prerandomization process, a cross-sectional study was conducted. We recruited children and adolescents aged 7 to 15 years from Bangkok, Thailand. Eating behaviors were assessed by the Children's Eating Behavior Questionnaire (CEBQ), which is a parent or self-reported research instrument conducted by face-to-face interviews. Body mass index (BMI), BMI-for-age Z-score, waist and hip circumferences, and body compositions were assessed. Pearson's correlation coefficients were used to assess associations between the study variables.

**Results:**

Ninety-seven Thai children and adolescents with obesity participated in the study; 59 (61%) were male. Median [IQR] of age and BMI z-score were 10.5 [9.0, 12.2] years and 3.0 [2.6, 3.7], respectively. Subscale for Enjoyment of Food had the highest score. There were no associations between eating behaviors and BMI z-score. However, Emotional Overeating was associated with fat-free mass index (correlation coefficient = 0.24, *p*=0.02) and girls with obesity had lower scores in “Slowness in Eating” compared to boys [mean 2.1 versus 1.8, 95% CI: (−0.06, −0.01), *p*=0.04].

**Conclusion:**

Among Thai children and adolescents with obesity, the difference in multidimensional eating behavior might be affected by fat-free mass. Additional study with a larger sample size needed to explore underlying mechanisms and findings can be used to develop future behavior modification program.

## 1. Introduction

Obesity is a major problem worldwide. In 2016, the World Health Organization (WHO) reported that there were over 340 million children and adolescents aged between 5 and 19 who were overweight or obese [1]. In Thailand, the prevalence of overweight and obesity among children and adolescents aged 6–14 years has increased dramatically from 5.8% in 1995 to 9.7% in 2009 [2] and the prevalence of overweight and obesity in boys has increased compared to girls [2]. WHO defines obesity as “abnormal or excessive accumulation of body fat that presents a risk to health [3].” Diseases related to obesity (i.e., diabetes, cardiovascular disease, and some forms of cancer) that were previously diagnosed in adults are now emerging in children [4].

It is agreed that the food intake of children and adolescents with obesity exceeds their energy expenditure. In addition, children with obesity show specific eating behaviors and have more responses to food cues when compared to children with normal weight. Eating behaviors are influenced by both internal and external factors including food availability, knowledge, attitude, emotional state, and experience of the individuals [5]. Genetic differences can influence a child's eating behavior and food preferences are developed during infancy. Moreover, exposure to foods, including parental feeding practices, can also contribute to a child's natural response to food and taste preferences [6]. It is accepted that the family (i.e., parents, siblings, other relatives, and even children of the same age) play a major role in food preference, eating behavior, and childhood obesity [7]. In addition, family socioeconomic status (SES) and parental education may contribute to the development of childhood obesity. A recent review showed that low SES families consume large amounts of unhealthy food and have poorer eating behaviors [8]. Different patterns of eating behaviors can influence weight gain. Schachter [9] showed that responsiveness to satiety was low in obese individuals which resulted in poor regulation of their energy intake and overeating behavior. Furthermore, eating speed is an important factor for developing adiposity [10]. It was found that children with obesity ate faster and failed to have normal patterns of slowing down towards the end of the meal. The authors suggested that this pattern could reflect an impaired response to satiety signals. Moreover, Fogel et al. [11] reported that children who ate faster had higher food intake, resulting in higher body mass index (BMI) z-score and adiposity.

Several psychometric instruments have been developed to detect individual differences in eating behaviors. The Children Eating Behavior Questionnaire (CEBQ) is an instrument widely used in different populations and age groups across the world. Several countries, including Thailand, have shown that this instrument is valid and reliable with good psychometric properties, internal consistency, and reproducibility [12–15]. The CEBQ is constructed to assess eight aspects of children's eating behavior. There are four subscales that measure food-approach behaviors (food responsiveness, enjoyment of food, emotional overeating, and desire to drink) and the remaining four subscales measure food-avoidant behaviors (satiety responsiveness, slowness in eating, emotional undereating, and food fussiness). Prior research has shown a significant association between adiposity and food-approach scales, such as enjoyment of food and food responsiveness, while decreased scores were found to be associated with food-avoidant behaviors [12, 16]. In addition, the CEBQ has been used in other studies comparing appetite preferences of children who had lean and obese parents, the continuity and stability of a child's eating behavior during childhood, and the relationship between temperament and eating behaviors in young children [17–19].

Despite this, several studies have examined the relationship between the eating behavior and adiposity in children with obesity. However, there is limited study of specific behaviors associated with obesity among Thai children and adolescents. Therefore, we aimed to examine the association between CEBQ scores and body composition of Thai children and adolescents with obesity.

## 2. Materials and Methods

### 2.1. Study Design and Study Participants

This prerandomized, cross-sectional analysis of the randomized trial study titled “Impact of Dietary Fiber as Prebiotics on Intestinal Microbiota in Obese Thai Children” (ClinicalTrial.gov NCT03968003, *n* = 165) recruited and enrolled children and adolescents with obesity, aged between 7 and 15 years, from the Nutrition Clinic and Pediatric Obesity Clinic at the King Chulalongkorn Memorial Hospital, Bangkok, Thailand. Obesity was defined as having a BMI z-score above 2 standard deviations (SDs) to 3 SDs. Severe obesity was defined as having a BMI z-score of more than 3 SDs, according to the WHO median growth reference [20]. Written informed consent was obtained from all participants and their parents/guardians. Assent forms were also obtained from children aged between 7 and 12 years. The study protocol was approved by the Medical Ethics Committee of the Research Affairs, Faculty of Medicine, Chulalongkorn University (IRB. No. 240/60).

### 2.2. Anthropometry and Body Composition

Anthropometric measurements were performed by trained personnel who instructed study participants to wear thin clothes and remain barefoot during the measuring process. Weight and height were measured in all participants to the nearest 0.1 kg and 0.1 cm, respectively. Waist (at the umbilicus) and hip (at the maximal circumference of the buttocks) were measured to the nearest 0.1 cm using narrow, flexible, and inelastic steel measuring tape. The participants were asked to breathe normally and stand with their weight evenly distributed while crossing their arms over their shoulders during the measurements. Two measurements were taken at each measurement site and if a difference of over 1 cm between the measurements occurred, a third measurement was taken. BMI was calculated as weight in kilograms divided by the square of height in meters (kg/m^2^). BMI-for-age z-score (BAZ) was calculated based on the WHO Reference 2007 using the WHO Anthroplus program [21]. Body composition was measured by bioelectrical impedance analysis using the InBody 770 (InBody Co., Ltd., Chungcheongnam-do, Korea). Sex- and age-adjusted fat mass index (FMI, fat mass (kg)/height (m)^2^), fat-free mass index (FFMI, fat-free mass (kg)/height (m)^2^), and trunk fat mass index (TFMI, trunk fat mass (kg)/height (m)^2^) [22] were also calculated.

### 2.3. Dietary Assessment

Dietary intake was assessed by a dietician via interview using the 24-hour dietary recall method. Daily calories, percentage of energy distributions (carbohydrate: protein: fat), and nutrient intake were calculated using the Institute of Nutrition, Mahidol University Calculation-Nutrients (INMUCALs) Version 3.0 which contains more than 2,000 food items from Thailand [23].

### 2.4. Psychometric Measurement

The original English version of the CEBQ was previously translated and validated into Thai by Sirirassamee and Hunchangsith [15]. The Thai version of the CEBQ has good internal reliability (Cronbach's *α* ranging between 0.64 and 0.80) and correlation between subscales corresponding to the original version [24]. The CEBQ consists of 35 items with eight subscales, each consisting of 3–6 items rated on a five-point Likert scale (1 = never to 5 = always). (1) The food responsiveness (FR) subscale has 5 items representing responses to environment food cues (e.g., “If given the chance, my child would always have food in his/her mouth”). (2) The satiety responsiveness (SR) subscale has 5 items that reflect decreased hunger caused by food consumption (e.g., “My child gets full before his/her meal is finished). (3) The emotional overeating (EOE) and (4) emotional undereating (EUE) subscales have 4 items each that reflect the fluctuation of eating in response to negative emotional contexts (e.g., “My child eats more when annoyed” and “My child eats less when upset,” respectively). (5) The enjoyment of food (EF) subscale has 4 items representing the desire to eat and pleasure eating the food (e.g., “My child enjoys eating”). (6) The desire to drink (DD) subscale has 3 items that reflect general tendencies to consume sugary beverages (e.g., “If given the chance, my child would always be having a drink”). (7) The slowness of eating (SE) subscale has 4 items that reflects reduction of speed while eating a meal or prolonged meal time (e.g., “My child eats more and more slowly throughout the course of the meal”) (8) The food fussiness (FF) subscale has 6 items that represents picky eating or limited variety of foods that are accepted (e.g., “My child decides that he/she does not like the food, even without tasting it”) [12, 25]. According to the instrument instructions, there are reverse scores for five items due to opposite phrasing. The CEBQ is a multidimensional parent-reported questionnaire that examines the eating behaviors of children below 12 years of age. In this study, we included adolescents who used a different questionnaire to assess obesity-related appetitive traits. The self-reported CEBQ for adolescents was developed based on the Loh et al. questionnaire [13]. For example, the third person pronoun “my child” (parent-reported) was transferred to the first person “I” (self-report) for all items. The questionnaire was reviewed and translated to Thai and then back to English by bilingual translators who were fluent in both languages and had extensive expertise in the field of child development. The adolescent CEBQ has 8 subscales and 35 items with a five-point Likert scale. In addition, there were face-to-face interviews with experts in the field to assess the clarity and the overall wording of the questionnaire followed by revisions of the questionnaire to maximize response and minimize respondent burden.

### 2.5. Statistical Analysis

The normality of the data was evaluated using the Shapiro–Wilk Test. Descriptive statistics were conducted with normally distributed data and presented as the mean and SD. Pearson Chi-square tests for independent samples (categorical variables) and *t*-tests for independent samples (continuous variables) were used to examine differences in CEBQ scores between obese and severely obese children. Pearson's correlation coefficients were used to evaluate the relationships between the CEBQ and dietary intake as well as CEBQ and body composition. Spearman's correlation coefficients were used for nonparametric data.

All statistical tests were 2-sided and a *p* value <0.05 was considered statistically significant. All data were analysed using Stata version 15.1 (Stata Statistical Software: Release 15, College Station, TX: StataCorp LLC. 2017).

## 3. Results

### 3.1. Participants' Demographic, Anthropometric, Dietary Assessment, and Their Eating Behavior Scores

**Figure 1:**
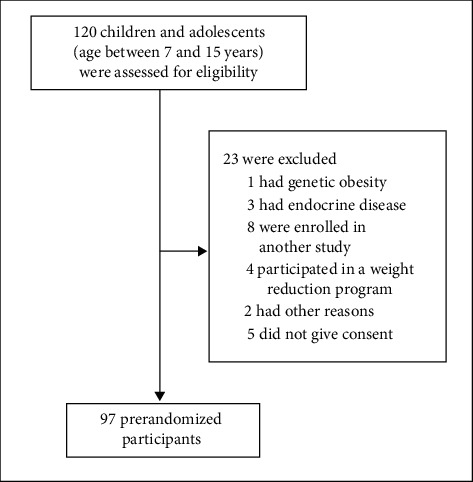
Recruitment flow chart.

A total of 97 prerandomized children and adolescents with obesity (median age 10.5 [9.0, 12.2] years, 61% male) participated in the study (Figure 1). General characteristics, anthropometry, body composition, and dietary assessment are shown in Table 1. The mean values for 4 out of 8 factors were above the scale midpoint (2.5). Overall, the mean scores for each individual's “food approach” scales were generally higher than the “food avoidant” scales except for food fussiness and emotional undereating which had a mean score that was higher than emotional overeating. From the four “food approach” subscales, the enjoyment of food subscale had the highest score. From the four “food avoidant” subscales, the food fussiness or picky eater had the highest score. In addition, there was no difference in eating behavior scores among children with obesity and severe obesity.

### 3.2. Sex and Age Differences

Eating behaviors in girls were significantly different compared to boys. It was found that girls ate their food slower according to the slowness in eating subscale compared to boys (mean = 2.1 versus 1.8, 95% CI: −0.66, −0.01), *p*=0.04). There were no significant differences between the mean values of eating behaviors among the ages of the participants ((children (age <12 y) versus adolescents (age ≥12 y)).

### 3.3. Correlations between CEBQs and Dietary Intake as Well as CEBQs and Body Compositions

Correlations between CEBQ subscales and variables are presented in Table 2. There was no association between eating behaviors and BAZ, whereas only the emotional overeating subscale showed a positive correlation between FFMI and eating behaviors.

## 4. Discussion

To the best of our knowledge, there is no clear association between CEBQ and BAZ in Thai children and adolescents with obesity. However, the appetitive profile of the children with obesity showed that the scores for “food-approach” were higher than the “food-avoidant” behaviors. Our findings are similar to many previous studies [25–27] which reported that children and adolescents with obesity exhibited weaker satiety responses and stronger appetitive responses to food as indicated by the scores from the CEBQ.

From our findings, we also observed that enjoyment of food had the highest score in the “food-approach” subscales. Enjoyment of food reflects a food liking behavior and a strong predictor of how likely it is to be consumed. Carnell and Wardle [28] found that there was an association between enjoyment of food (i.e., liking) and food responsiveness (i.e., wanting and salience) with a faster rate of eating and greater overall caloric intake. Children who have more enjoyment of food are also more likely to consume greater quantities of food despite being satiated. Moreover, higher enjoyment of food among those children who were overweight and obese was associated with a higher frequency to snack on foods that have high energy and high calories unlike normal weight children [29]. The current study findings suggest that high enjoyment of food in children with obesity may contribute as factors affecting childhood obesity.

On the other hand, the food fussiness subscale had the highest “food-avoidant” behavior scores. A recent research [30] revealed that food fussiness may play a role in pediatric obesity development and maintenance. Food fussiness or picky eater refers to a child's unwillingness to eat specific foods or try new foods, resulting in consumption of a limited variety of food types or food items. Food fussiness typically emerges at the age of six and it is relatively stable through childhood. Food fussiness has been associated with children's lower intake of vitamins, minerals, dietary fiber, and certain healthy food groups such as vegetables [30, 31]. Evidence regarding the effects of picky eating on childhood obesity is still controversial as picky eaters tend to have lower weight and reduced risk of obesity [32]. However, picky eating also exists in children and adolescents with overweight and obesity. Picky eaters may compensate their limited intake of disliked food with a higher intake of more palatable energy-dense food [30]. Therefore, this may increase the risk of the child becoming obese. Furthermore, children with high food fussiness have been shown to consume more sweetened foods than children with low food fussiness behavior [33].

Previous studies have shown a negative association between satiety response with BMI and waist circumference [12, 26]. In contrast, we could not find any association between CEBQ and BMI even though other body composition parameters were measured. We found that emotional overeating was significantly positively correlated with FFMI. This finding is consistent with prior research that showed a bidirectional association between BMI and emotional overeating [34]. Past research has concluded that emotional overeating resulted in more food intake and weight gain, while, in turn, excess weight may lead to overeating (e.g., due to higher body dissatisfaction). In addition, Steinbekk et al. [35] showed that higher muscle mass was associated with decreased satiety responsiveness and higher fat mass could predict a greater increase in food responsiveness. This finding suggests that children with higher fat mass are more likely to eat in response to external food stimuli, whereas children with higher FFM are more likely to eat even when they are full. This observation is supported by the study conducted by Blundell et al. [36] in which they demonstrated that adults with obesity had a greater amount of FFM consumed larger proportions of food with high energy. One possible explanation is that skeletal muscles could send signals to the central nervous system that controls the energy intake [37]. Aside from the muscle mass impacting the homeostatic appetite control system, it is also responsible for resting energy expenditure to maintain the body's lean tissues and all vital organs [38]. It is expected that obese individuals have a persistent drive to eat because they have a larger FFM and increased adipose tissue which results in a higher resting metabolic rate than smaller people or individuals who are lean. Future research is needed to identify the body composition affected by the eating behavior of children and adolescents with obesity.

The comparison of CEBQ between Thai children with obesity and severe obesity showed that there were no significant differences. Our findings contradict results reported from previous studies because the study population was different. The other studies [12, 25–27] included children with underweight, normal weight, and obesity. For example, Sanchez et al. [25] conducted a cross-sectional study in 1,058 children aged between 7 and 10 years. They excluded children with severe obesity, whereas, in our study, we included both children with obesity and severe obesity. Therefore, the magnitude of the correlation may be too subtle to be statistically significant.

Furthermore, child gender showed differences in the speed of eating between girls and boys. We found that girls with obesity were slower eaters compared to boys. This finding is in line with Ochiai et al. [39] who reported that girls ate slower than boys after the OR was adjusted for birth weight, parental obesity, exercise, and skipping breakfast in children with overweight. Likewise, Hill and McCutcheon [40] showed that men ate faster than women. The reason why boys ate much faster than girls may be due to the food preference. The girls preferred eating fruits and vegetables compared to boys who preferred fatty and sugary foods, meats, eggs, and processed meat products [41]. On the contrary, Gross et al. [42] reported that sex did not affect the children's eating behavior. As a result of these conflicting findings, future studies should assess eating behaviors and biological mechanisms between the sexes.

Strengths of this study include the fact that this is the first study to explore eating behaviors and the association between CEBQ and body compositions in Thai children and adolescents with obesity. We used the original CEBQ that was translated and validated in Thai children and pioneered the first adaptation of the CEBQ as a self-reported questionnaire to assess the association of eating behaviors and its correlation among Thai adolescents with obesity. However, there were some limitations. First, there was an inherent uncertainty related to the subjective measurement of eating behaviors in children because the questionnaires were completed by the parents. We did not assess the parental food preferences and their eating styles. Second, this was a cross-sectional study with a small sample size. Third, we did not have any information on other variables that might affect the children's eating behaviors such as parental characteristics and education level, socioeconomic status, and family environment. Thus, unmeasured potential confounders cannot be ruled out.

## 5. Conclusions

Our findings revealed that the differences in body composition include fat-free mass and gender which affected eating behaviors in Thai children and adolescents with obesity. Additional longitudinal studies are warranted to assess eating behaviors among children with obesity, etiology of adiposity, and satiety mechanisms.

## Figures and Tables

**Table 1:** Demographic data, body composition, dietary assessment, and CEBQ scores of the participants.

	Obese children and adolescents (*n* = 97) (means ± SD or median [IQR])
Age (years)	10.5 [9.0, 12.2]
Sex, male (*n*, %)	59 (61%)
Body weight (kg)	62.8 ± 16.6
Height (cm)	147.7 ± 12.2
BMI (kg/m^2^)	27.8 [25.5, 30.5]
BMI-for-age z-score (BAZ)	3.0 [2.6, 3.7]
Waist circumference (cm)	90.5 ± 10.3
Hip circumference (cm)	95.9 ± 11.1

*Body impedance analysis (BIA)*	
Fat mass (kg)	26.0 ± 8.1
Fat mass index (kg/m^2^)	11.7 ± 2.7
Fat-free mass (kg)	34.9 [29.2, 42.2]
Fat-free mass index (kg/m^2^)	15.8 [14.8, 17.3]
Skeletal muscle mass (kg)	18.5 [15.3, 22.9]
Percent body fat (%)	42.5 [37.6, 45.4]
Trunk fat mass (kg)	12.6 ± 4.0
Trunk fat mass index (kg/m^2^)	5.7 ± 1.4

*Total nutrients intake*	
Caloric intake (kcal/day)	1446.3 [1156.9, 1916.0]
Protein intake (g/kg/day)	1.1 [0.8, 1.3]
Cholesterol intake (mg/day)	331.1 [133.2, 535.4]
Energy distribution (C : P : F) (%)	48 : 17 : 35

*Eating Behaviors Scores*	
Food Responsiveness (FR)	3.2 ± 0.9
Enjoyment of Food (EF)	4.1 ± 0.7
Emotional Overeating (EOE)	2.1 ± 0.9
Desire to Drink (DD)	3.1 ± 1.2
Satiety Responsiveness (SR)	2.1 ± 0.6
Slowness in Eating (SE)	1.9 ± 0.8
Emotional Undereating (EUE)	2.4 ± 0.8
Food Fussiness (FF)	2.6 ± 0.9

Energy distribution (carbohydrate : protein : fat), expressed as percentage.

**Table 2:** Correlations between CEBQ subscales and variables (*n* = 97).

Subscales	BAZ (95% CI)	WC (95% CI)	FMI (95% CI)	FFMI (95% CI)	TFMI (95% CI)
*Food approach*					
Food responsiveness (FR)	−0.01	0.04	0.03	−0.07	0.06
	(−0.18, 0.19)	(−0.19, 0.28)	(−0.18, 0.23)	(−0.25, 0.14)	(−0.15, 0.27)
Enjoyment of food (EF)	0.05	0.01	−0.02	−0.15	0.03
	(−0.12, 0.22)	(−0.20, 0.23)	(−0.24, 0.20)	(−0.32, 0.04)	(−0.19, 0.24)
Emotional overeating (EOE)	−0.01	0.17	0.05	0.24^*∗*^	0.06
	(−0.19, 0.22)	(−0.04, 0.37)	(−0.13, 0.27)	(0.01, 0.42)	(−0.12, 0.27)
Desire to drink (DD)	−0.03	−0.02	−0.14	0.04	−0.12
	(−0.24, 0.15)	(−0.20, 0.17)	(−0.33, 0.05)	(−0.13, 0.22)	(−0.31, 0.07)

*Food avoidant*					
Satiety responsiveness (SR)	0.09	−0.18	0.01	−0.13	−0.01
	(−0.13, 0.28)	(−0.35, 0.02)	(−0.20, 0.20)	(−0.29, 0.04)	(−0.20, 0.19)
Slowness in eating (SE)	0.10	−0.01	0.12	0.13	0.08
	(−0.09, 0.27)	(−0.23, 0.20)	(−0.12, 0.33)	(−0.12, 0.35)	(−0.17, 0.30)
Emotional undereating (EUE)	0.02	0.01	−0.01	−0.05	−0.02
	(−0.17, 0.23)	(−0.19, 0.20)	(−0.19, 0.19)	(−0.22, 0.13)	(−0.21, 0.17)
Food fussiness (FF)	0.10	−0.12	0.01	−0.18	−0.01
	(−0.08, 0.25)	(−0.31, 0.08)	(−0.20, 0.20)	(−0.36, 0.02)	(−0.21, 0.19)

^^*∗*^^ *P* value <0.05; BAZ, BMI-for-age z-score; WC, waist circumference; FMI, fat mass index; FFMI, fat-free mass index; TFM, trunk fat mass; TFMI, trunk fat mass index.

## Data Availability

The present study data used to support the findings of this study are available from the corresponding author upon request.
